# Supplementary material for: Inherent potential of steelmaking to contribute to decarbonisation targets via industrial carbon capture and storage
Source: Nat Commun. 2018 Oct 24;9:4422. doi: 10.1038/s41467-018-06886-8 (PMC6200798; doi:10.1038/s41467-018-06886-8)
Supplement: Supplementary file 1 — Supplementary Information [file 41467_2018_6886_MOESM1_ESM.pdf]

Supplementary Information for

Inherent potential of steelmaking to contribute to decarbonisation targets via industrial carbon capture and storage

Tian et al.

This PDF file includes:

Supplementary Figures 1-8

Supplementary Tables 1-4

Supplementary Notes 1-6

Supplementary References 58-65

Supplementary Figures

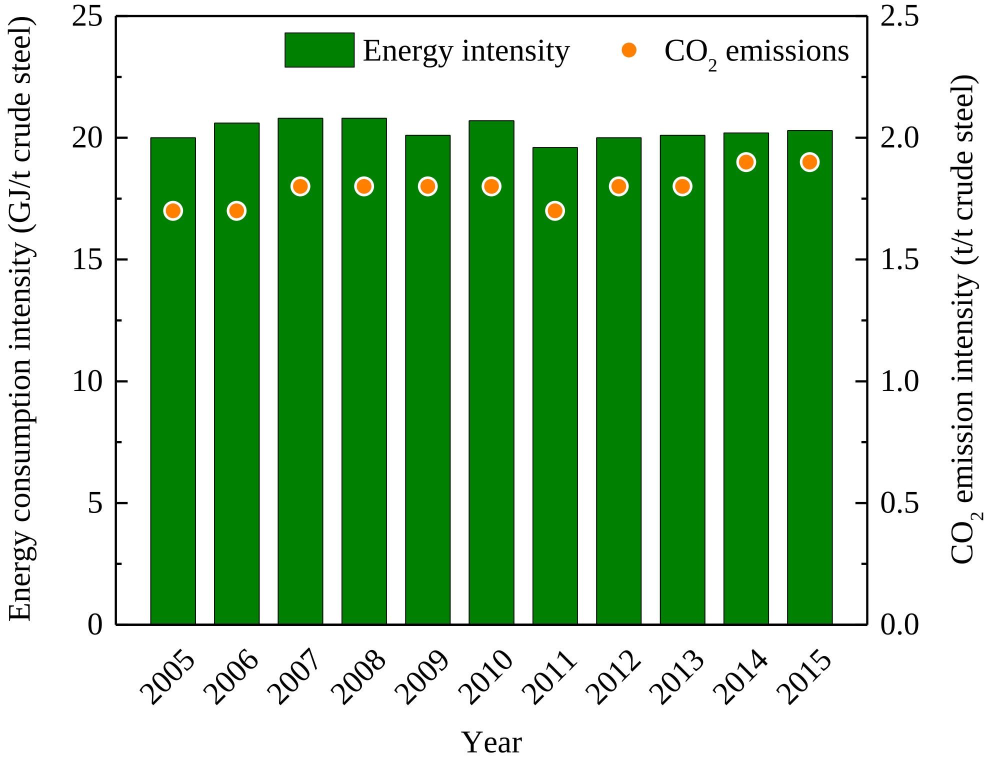

Supplementary Figure 1. Intensity of energy consumption and CO_2 emission of global iron and steel industry from 2005 to 2015[7,9,10].

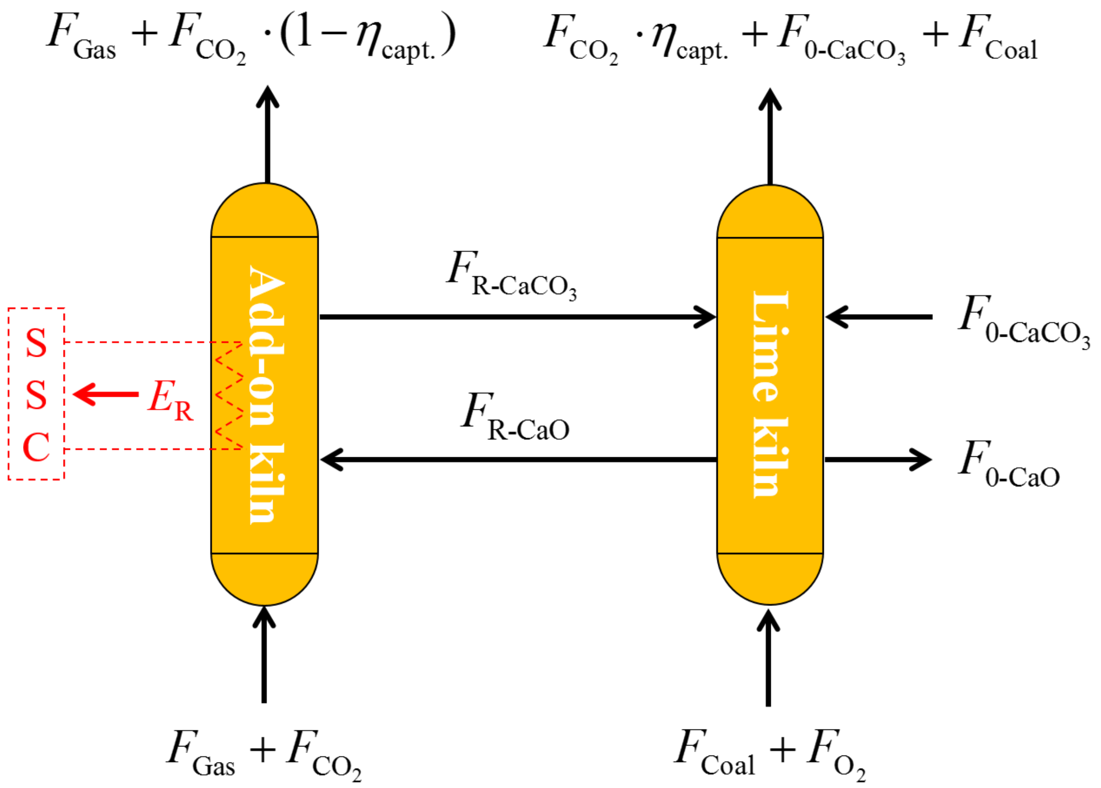

Supplementary Figure 2. Process flow diagram of the calcium-looping lime production (CaL-LP) scheme. *F* - molar flow rate of different substances involved, kmol/s; $\eta_{capt.}$ - CO_2 capture efficiency in the add-on kiln, %; E_R - heat recovered from the add-on kiln, GJ/t crude steel; and SSC - secondary steam cycle for electricity generation.

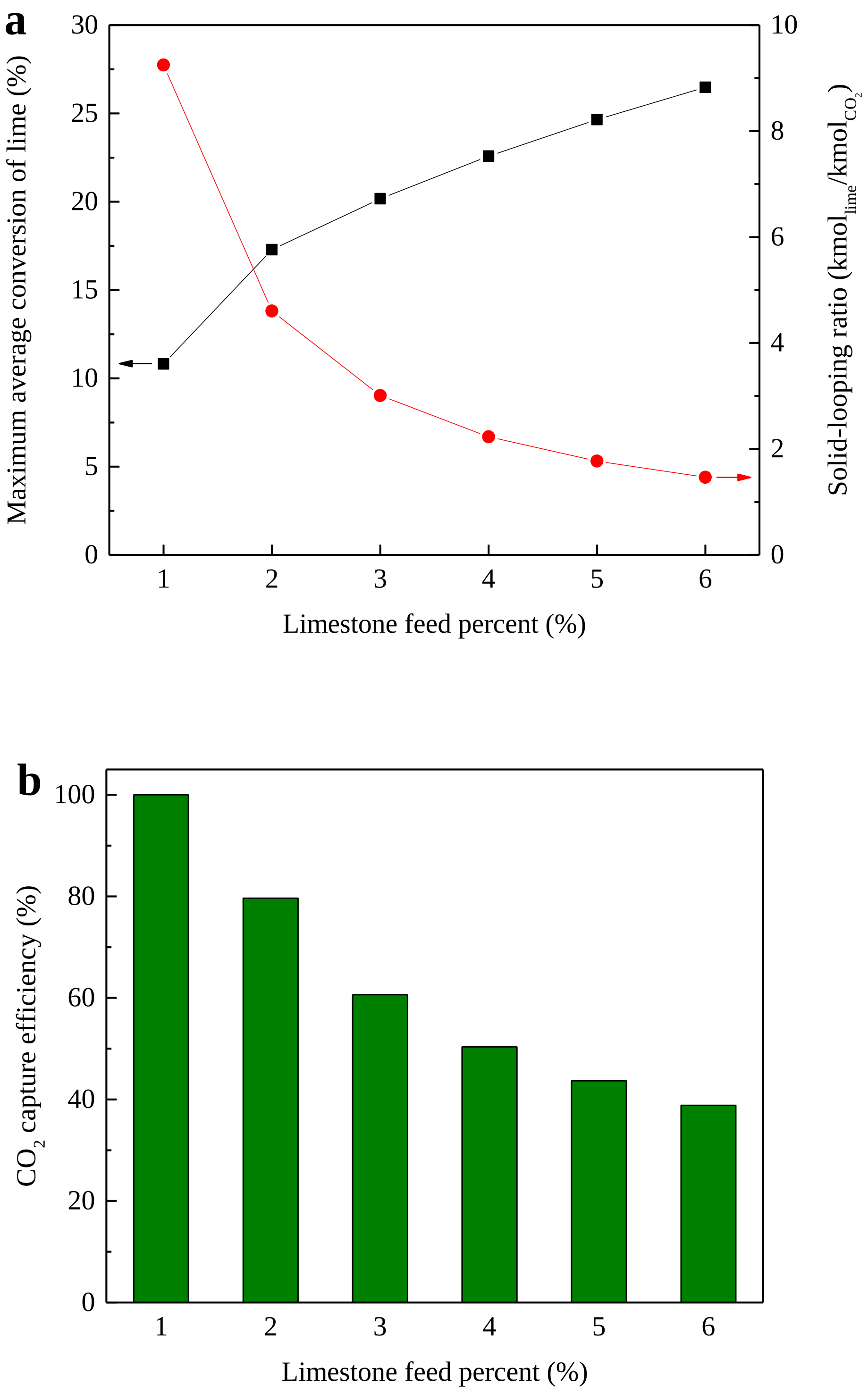

Supplementary Figure 3. Process simulation of CO_2 capture in the add-on kiln. (**a**) Maximum average conversion of lime and solid-looping ratio, and (**b**) CO_2 capture efficiency as a function of the limestone feed percent during the calcium-looping lime production process proposed for decarbonisation of the iron and steel industry.

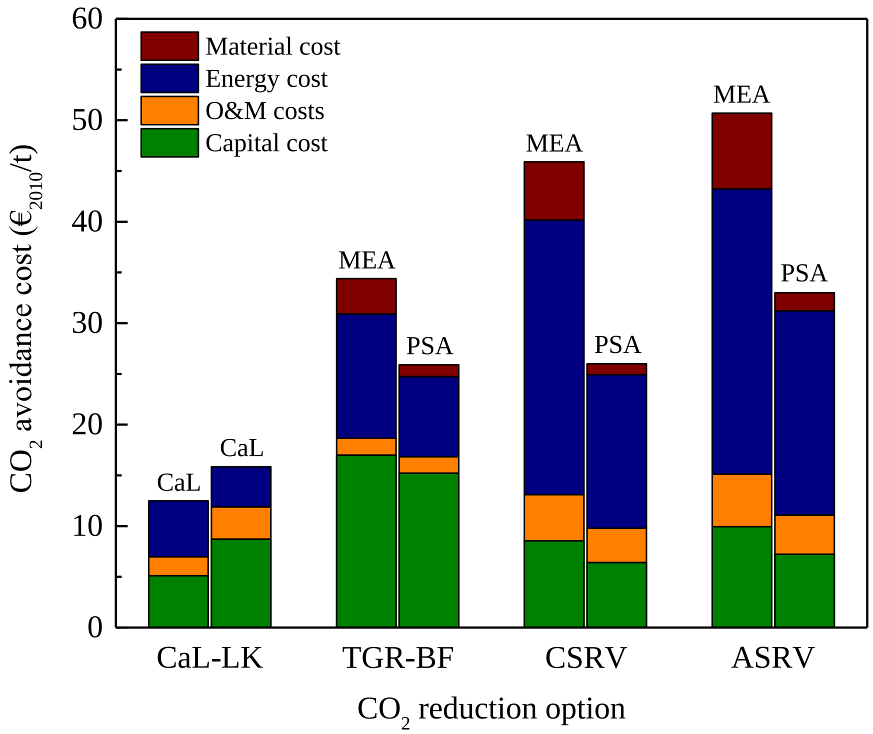

Supplementary Figure 4. Breakdown of the CO_2 avoidance cost of main decarbonisation options for the iron and steel industry. A comparison of the cost structure between the CaL-LP scheme and other options developed for CO_2 reduction in different steelmaking processes. The iron and steel production facilities: CaL-LK - calcium-looping lime kiln, TGR-BF - top gas recycling-blast furnace, CSRV - conventional smelting reduction vessel, and ASRV - advanced smelting reduction vessel; and the CO_2 capture technologies: CaL - calcium looping, MEA - amine scrubbing using monoethanolamine, and PSA - pressure swing adsorption.

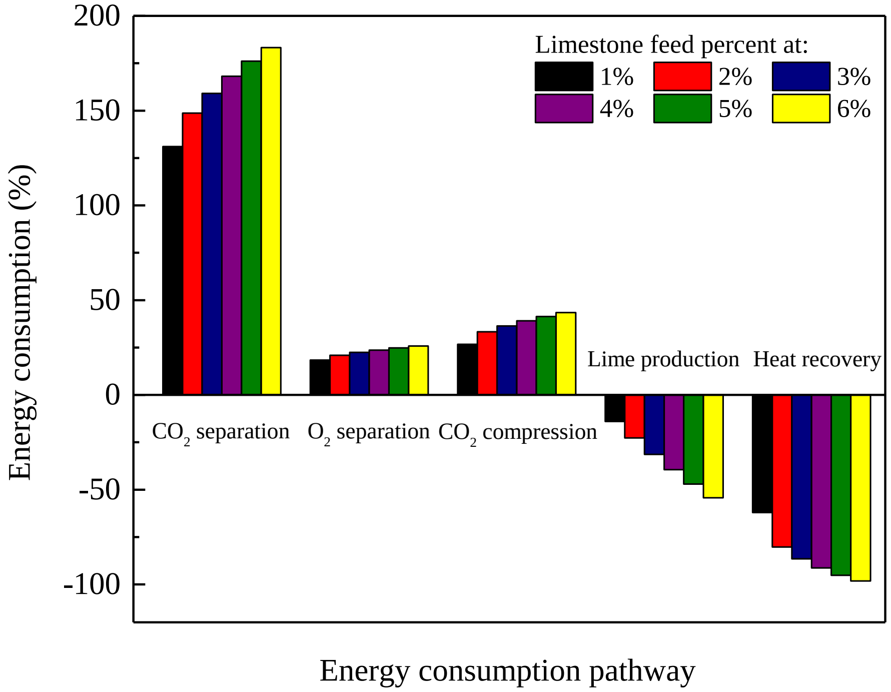

Supplementary Figure 5. Breakdown of the primary energy consumption of the CaL-LP scheme. Consumption structure of the incremental primary energy due to implementation of the CaL-LP scheme at different limestone feed percents for CO_2 emission reduction in an integrated steel mill.

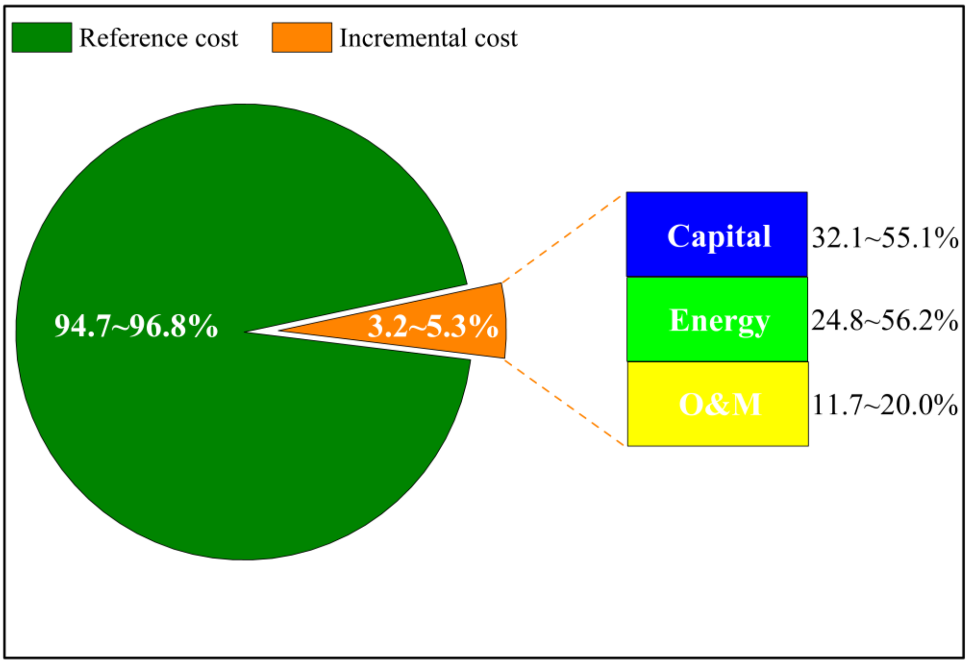

Supplementary Figure 6. Overall impact of the CaL-LP scheme on steelmaking economics. Composition of the incremental cost due to implementation of the CaL-LP scheme for CO_2 reduction in an integrated steel mill and its contribution percentage to the overall manufacturing cost of crude steel.

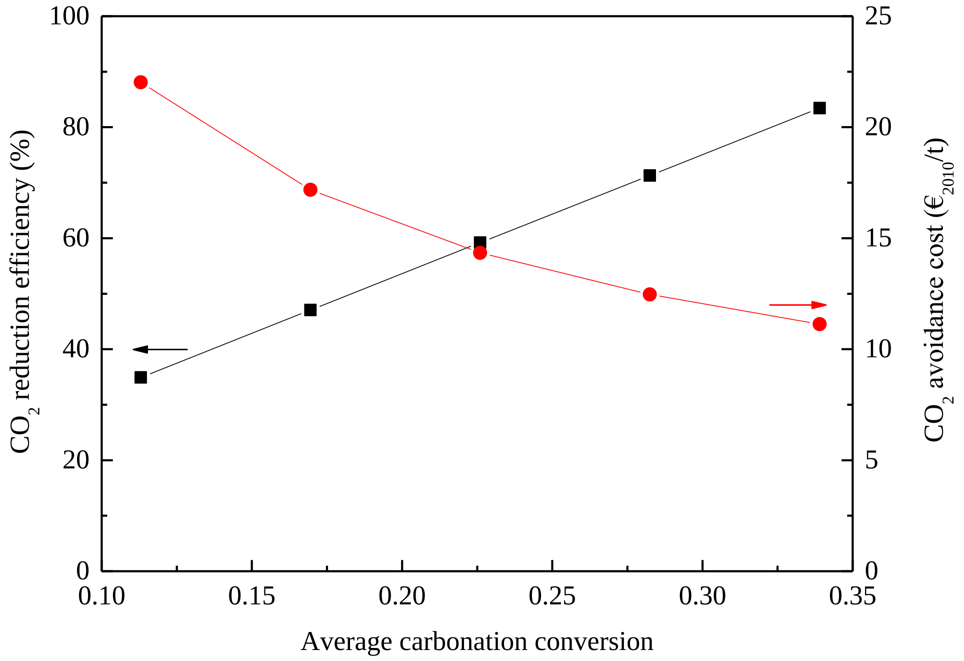

Supplementary Figure 7. CO_2 reduction efficiency and avoidance cost as a function of the average carbonation conversion of lime in the CaL-LP scheme.

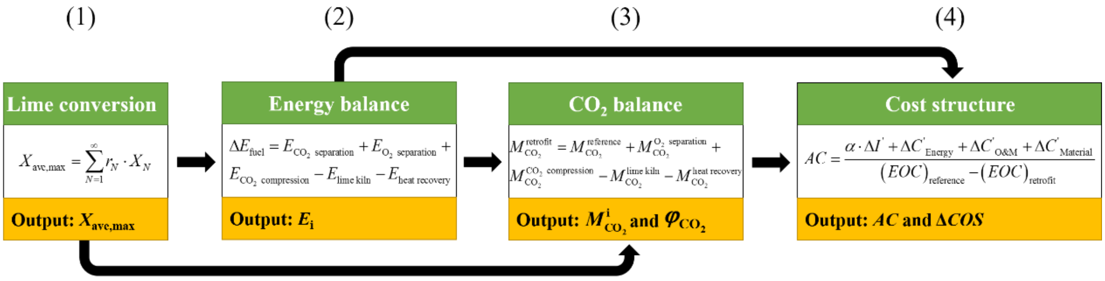

$X_{ave,max}$: the maximum average carbonation conversion of lime in the add-on kiln.

r_N: the mass fraction of the lime particles that has experienced N carbonation-calcination cycles.

X_N: the carbonation conversion of the lime particles that has experienced N carbonation-calcination cycles.

ΔE_{fuel}: the total incremental primary energy consumption due to implementation of the process retrofitting scheme.

E_i: the energy required or recovered from related sub-sectors in a conventional integrated steel mill.

$M_{CO_2}^{i}$: total amount of CO_2 generated in the retrofit and reference steel mill, respectively, and the amount of CO_2 generated or avoided from related sub-sectors in the steel mill.

φ_{CO_2}: overall CO_2 reduction potential of the steel mill retrofitted with calcium-looping lime production scheme.

AC: CO_2 avoidance cost of the steel mill retrofitted with calcium-looping lime production scheme.

$\Delta I'$, $\Delta C'_{Energy}$, $\Delta C'_{O\&M}$, $\Delta C'_{Material}$: the additional capital requirement, and annual cost of energy, O&M, and raw material, respectively, of the steel mill implemented with calcium-looping lime production.

EOC: the emission of CO_2 per tonne of steel produced.

ΔCOS: the incremental levelized cost of steel.

Supplementary Figure 8. Methodology framework to evaluate the techno-economics of implementing the CaL-LP scheme for CO_2 reduction in the iron and steel industry.

Supplementary Tables

Supplementary Table 1. Semi-empirical correlations commonly considered for characterisation of the carbonation conversion of CaO-based CO_2 sorbents.

Equation	Correlation [a]	Parameter [b]	X_N for lime [c]	Data source
S1	$X_N = k^{N+1} + X_r$	k=0.782, X_r=0.174	0.174	Abanades[48]
S2	$X_N = \frac{1}{\frac{1}{1-X_r} + k \cdot N} + X_r$	k=0.52, X_r=0.075	0.094	Grasa and Abanades[58]
S3	$X_N = a_1 \cdot f_1^{N+1} + a_2 \cdot f_2^{N+1} + X_r$	a_1=1.0, a_2=0, f_1=0.712, f_2=0, X_r=0.074	0.074	Li et al.[59]
S4	$X_N = f_m^N \cdot (1 - f_w) + f_w$	f_m=0.77, f_w=0.17	0.170	Abanades and Alvarez[60]
S5	$X_N = \frac{1}{1 + k \cdot N}$	k=0.06	0.143	Wang and Anthony[61]

[a], X_N denotes the carbonation conversion achieved after N cycles of carbonation–calcination, k denotes the deactivation constant, X_r denotes the residual carbonation conversion, a_1, a_2, f_1, f_2, f_m, and f_w are the fitting parameters; [b], the corresponding values when the CaO-based CO_2 sorbents are natural limestones; [c], the carbonation conversion achieved after 100 cycles of carbonation–calcination.

Supplementary Table 2. Key parameters employed in the thermodynamic simulation of the calcium-looping lime production process.

Parameter	Unit	Value	Data source
Carbonation temperature ($T_{car.}$)	K	923.15	Hanak et al.[18]
Calcination temperature ($T_{cal.}$)	K	1173.15	Hanak et al.[18]
Molar heat capacity (CP_{CaCO_3}) [a]	cal/(mol·K)	[24.98; 5.24; -6.20] [b]	Barin and Knacke[51]
Molar heat capacity (CP_{CaO})	cal/(mol·K)	[11.86; 1.08; -1.66]	Barin and Knacke[51]
Molar heat capacity (CP_{CO_2})	cal/(mol·K)	[10.55; 2.16; -2.04]	Barin and Knacke[51]
Molar heat capacity (CP_{O_2})	cal/(mol·K)	[7.16; 1.00; -0.40]	Barin and Knacke[51]
Molar heat capacity (CP_{N_2})	cal/(mol·K)	[6.66; 1.02; 0]	Barin and Knacke[51]
Carbonation enthalpy ($\triangle H_{car.}$) [c]	kJ/mol	-171.618	Barin and Knacke[51]
Calcination enthalpy ($\triangle H_{cal.}$) [d]	kJ/mol	166.042	Barin and Knacke[51]
Fuel heat value (HV_{ce}) [e]	MJ/kg	29.3	IEA[52]
Mass ratio ($FR_{CO_2/limestone}$) [f]	kg/kg	6.0	WSA[16]
Mass feed ratio ($FR_{O_2/ce}$) [g]	kg/kg	2.67	Calculated

[a], CP_i denotes the molar heat capacity of substance i; [b], A, B, and C in the matrix "[A; B; C]" are the constants required to determine CP via the equation $CP = A + B \cdot 10^{-3} T + C \cdot 10^{5} T^{-2}$; [c], $\triangle H_{car.}$ denotes enthalpy of the carbonation reaction $CaO + CO_2 \rightleftharpoons CaCO_3$ at 900 K; [d], $\triangle H_{cal.}$ denotes enthalpy of the calcination reaction $CaCO_3 \rightleftharpoons CaO + CO_2$ at 1200 K; [e], HV_{ce} denotes the heat value of coal equivalent (ce); [f], $FR_{CO_2/limestone}$ denotes the mass ratio of the total CO_2 emitted to the limestone feedstock consumed in a conventional integrated steel mill; [g], $FR_{O_2/ce}$ denotes the mass feed ratio of O_2 to coal equivalent (ce) in the lime kiln of the calcium-looping lime production scheme.

Supplementary Table 3. Mass balance of the calcium-looping lime production process.

Limestone feed percent (%)	M_{ce} [a] (kg/t)	M_{O_2} [b] (kg/t)	$M_{CO_2\ captured}$ [c] (kg/t)	$M_{CO_2\ separated}$ [d] (kg/t)
1	400.8	1068.8	1427.1	2577.4
2	281.5	750.6	1141.2	1988.2
3	217.2	579.3	887.4	1571.1
4	182.6	487.0	745.2	1341.1
5	160.5	428.0	651.6	1191.3
6	144.9	386.4	583.2	1083.3

[a], the mass of coal equivalent input into the lime kiln per tonne of crude steel produced; [b], the mass of O_2 input into the lime kiln per tonne of crude steel produced; [c], the mass of CO_2 captured in the add-on kiln per tonne of crude steel produced; [d], the mass of CO_2 separated from the whole calcium-looping lime production process per tonne of crude steel produced.

Supplementary Table 4. Main techno-economic assumptions employed in this study.

Parameter	Unit	Value	Data source
Economic lifetime	yr	25	Ho et al.[40]
Project interest rate	%	10	Kuramochi et al.[50]
Financial year		2010	Tsupari et al.[28]
Primary energy price (*EP*)	€$_{2010}$/GJ	1.5	IEA[36]
CO_2 emission factor (ε) ^{a}	t/t	2.54	Tu and Liu[62]
CO_2 compression energy ($\overline{E}_{comp.}$)	kWh/t	110.9	Damen et al.[53]
Air separation energy ($\overline{E}_{sep.}$)	kWh/t O_2	184.8	Ströhle et al.[54]
Heat-electricity efficiency (δ) ^{b}	%	43.0	Zhao et al.[63] and Abanades et al.[64]
O&M costs ^{c}	%-capital cost	4	Kreutz et al.[56]
Add-on kiln cost ^{d}	€$_{2010}$/t crude steel	45	IEA[55]
Steam turbine cost	€$_{2010}$/t crude steel	25	IEA[55]

^{a}, amount of CO_2 emitted per tonne of coal equivalent consumed; ^{b}, the efficiency of steam turbine to transform heat into electricity; ^{c}, operation and maintenance costs of the retrofitting project; ^{d}, capital cost of the add-on kiln and CO_2 compressor, including engineering, procurement, and installation.

Supplementary Notes

Supplementary Note 1

Various semi-empirical correlations have been developed to characterise the carbonation conversion of naturally-derived or synthetic CaO-based sorbents for CO_2 capture up to now. Most of these correlations have been reviewed by Dean et al.[65] and Hanak et al.[18], and five most important ones are presented and compared here in Supplementary Table 1. For natural limestones, the correlation proposed by Grasa and Abanades (Supplementary Equation S2) gives a best estimate of the carbonation conversion, which is neither that optimistic as Supplementary Equations S1, S4, and S5, nor that pessimistic as Supplementary Equation S3. Therefore, Supplementary Equation S2 is employed in this study to characterise the cyclic carbonation conversion of lime derived from limestone calcination in the lime kiln.

Based on the assumption that the solids are well mixed in both the lime and add-on kilns, r_N, representing the mass fraction of the lime particles that enters the add-on kiln in the solid stream (F_R) and has been circulated N times through the loop described in Supplementary Figure 2, can be calculated from a succession of mass balances (Supplementary Equation S6)[48].

$$r_N = \frac{F_0 \cdot F_R^{N\text{-}1}}{\left(F_0 + F_R\right)^N} \qquad \text{(S6)}$$

If we define limestone feed percent (f_p, %) as the molar percent of the limestone freshly fed into the lime kiln (F_0, kmol/s) to that delivered from the add-on kiln (F_R, kmol/s), r_N can be expressed as a function of f_p (Supplementary Equation S7) via a mathematical transformation.

$$r_N = \frac{f_p}{\left(f_p + 1\right)^N} \qquad \text{(S7)}$$

Upon determination of $X_{ave,max}$, CO_2 capture efficiency ($\eta_{capt.}$, %) in the add-on kiln of the CaL-LP scheme (Supplementary Figure 2) can be described as:

$$\eta_{capt.} = \frac{F_R \cdot X_{ave,max}}{F_{CO_2}} \times 100\% = R \cdot X_{ave,max} \times 100\% \qquad \text{(S8)}$$

where the solid-looping ratio (R) is defined as the molar ratio of the lime circulated into the add-on kiln (F_R, kmol/s) to the inflow CO_2 in the flue gas (F_{CO_2} , kmol/s), and can be calculated according to Supplementary Equation S9.

$$R = \frac{F_{R}}{F_{CO_2}} = \frac{MW_{CO_2}}{f_{p} \cdot FR^{'}_{CO_2/limestone} \cdot MW_{CaCO_3}} \qquad (S9)$$

In Supplementary Equation S9, MW_{CO_2} and MW_{CaCO_3} denotes the molecular weight of CO_2 (44 kg/kmol) and $CaCO_3$ (100 kg/kmol), respectively, and $FR^{'}_{CO_2/limestone}$ denotes the mass feed ratio of CO_2 to limestone for the calcium-looping lime production process.

Supplementary Note 2

All terms in the equation $E_{CO_2\ \text{separation}} = E_{\text{cycled solid}} + E_{\text{fresh solid}} + E_{O_2} + E_{\text{reaction}}$ have the unit gigajoules per tonne of crude steel produced (GJ/t), and can be calculated via the following Supplementary Equations S10-S14, respectively.

$$E_{CO_2\ \text{separation}} = HV_{\text{ce}} \cdot M_{\text{ce}} \qquad \text{(S10)}$$

$$E_{\text{cycled solid}} = \int_{T_{\text{car.}}}^{T_{\text{cal.}}} \frac{M_{\text{limestone}}}{MW_{CaCO_3} \cdot f_{\text{p}}} \cdot \left[X_{\text{ave,max}} \cdot CP_{CaCO_3}(T) + \left(1 - X_{\text{ave,max}}\right) \cdot CP_{CaO}(T) \right] \cdot \mathrm{d}T \qquad \text{(S11)}$$

$$E_{\text{fresh solid}} = \int_{T_0}^{T_{\text{cal.}}} \frac{M_{\text{limestone}}}{MW_{CaCO_3}} \cdot CP_{CaCO_3}(T) \cdot \mathrm{d}T \qquad \text{(S12)}$$

$$E_{O_2} = \int_{T_0}^{T_{\text{cal.}}} \frac{M_{O_2}}{MW_{O_2}} \cdot CP_{O_2}(T) \cdot \mathrm{d}T \qquad \text{(S13)}$$

$$E_{\text{reaction}} = \left(X_{\text{ave,max}} + f_{\text{p}} \right) \cdot \frac{M_{\text{limestone}}}{MW_{CaCO_3} \cdot f_{\text{p}}} \cdot \Delta H_{\text{cal.}} \qquad \text{(S14)}$$

where $M_{\text{limestone}}$ denotes the average consumption of limestone per tonne of crude steel produced in a conventional integrated steel mill, and is valued at 300 kg/t in this study[16]; T_0 denotes the ambient temperature (298.15 K); and MW_{CaCO_3} and MW_{O_2} denote the molecular weight of $CaCO_3$ (100 kg/kmol) and O_2 (32 kg/kmol), respectively. The meaning and valuation of other parameters in Supplementary Equations S10-S14 are provided in Supplementary Tables 2 and 3.

Supplementary Note 3

Since $E_{CO_2\text{ separation}}$ has been determined above in Supplementary Note 2, other terms (gigajoules per tonne of crude steel produced, GJ/t) in the equation $\Delta E_{\text{fuel}} = E_{CO_2\text{ separation}} + E_{O_2\text{ separation}} + E_{CO_2\text{ compression}} - E_{\text{lime kiln}} - E_{\text{heat recovery}}$ can be calculated via the following Supplementary Equations S15-S18, respectively.

$$E_{\text{heat recovery}} = \eta_{\text{capt.}} \cdot \frac{M_{CO_2}^{\text{retrofit}}}{MW_{CO_2}} \cdot \Delta H_{\text{car.}} \qquad \text{(S15)}$$

$$E_{O_2\text{ separation}} = \overline{E}_{\text{sep.}} \cdot M_{O_2} \cdot \frac{1}{\delta} \qquad \text{(S16)}$$

$$E_{CO_2\text{ compression}} = \overline{E}_{\text{comp.}} \cdot M_{CO_2\text{ separated}} \cdot \frac{1}{\delta} \qquad \text{(S17)}$$

$$E_{\text{lime kiln}} = E_{\text{limestone}} + E_{\text{air}} + E_{\text{decomposition}} \qquad \text{(S18)}$$

where MW_{CO_2} denotes the molecular weight of CO_2 (44 kg/kmol), and the meaning and valuation of other parameters in Supplementary Equations S15-S17 are provided in Supplementary Tables 2-4. While in the conventional lime kiln (Supplementary Equation S18), the total primary energy consumption per tonne of crude steel produced ($E_{\text{lime kiln}}$) is made up of three parts; i.e., the energy required to heat limestone to the calcination temperature ($E_{\text{limestone}}$, GJ/t), to heat air to the calcination temperature (E_{air}, GJ/t), and to drive the endothermic limestone calcination (decomposition) reaction ($E_{\text{decomposition}}$, GJ/t). The value of $E_{\text{limestone}}$, E_{air}, and $E_{\text{decomposition}}$ can be calculated via the following Supplementary Equations S19-S21, respectively, with the parameters required for calculation provided in Supplementary Table 2.

$$E_{\text{limestone}} = \int_{T_0}^{T_{\text{cal.}}} \frac{M_{\text{limestone}}}{MW_{CaCO_3}} \cdot CP_{CaCO_3}(T) \cdot \mathrm{d}T \qquad \text{(S19)}$$

$$E_{\text{air}} = \int_{T_0}^{T_{\text{cal.}}} [\frac{M_{O_2}}{MW_{O_2}} \cdot CP_{O_2}(T) + \frac{78\%}{21\%} \cdot \frac{M_{O_2}}{MW_{O_2}} \cdot CP_{N_2}(T)] \cdot \mathrm{d}T \qquad \text{(S20)}$$

$$E_{\text{decomposition}} = \frac{M_{\text{limestone}}}{MW_{CaCO_3}} \cdot \Delta H_{\text{cal.}} \qquad \text{(S21)}$$

Supplementary Note 4

All terms in the equation $M_{CO_2}^{retrofit} = M_{CO_2}^{reference} + M_{CO_2}^{O_2\ separation} + M_{CO_2}^{CO_2\ compression} - M_{CO_2}^{lime\ kiln} - M_{CO_2}^{heat\ recovery}$ have the unit kilograms per tonne of crude steel produced (kg/t), and related terms in this equation can be calculated via Supplementary Equation S22:

$$M_{CO_2}^{i} = \frac{E_i}{HV_{ce}} \cdot \varepsilon \qquad (S22)$$

where $M_{CO_2}^{i}$ denotes the terms $M_{CO_2}^{O_2\ separation}$, $M_{CO_2}^{CO_2\ compression}$, $M_{CO_2}^{lime\ kiln}$, and $M_{CO_2}^{heat\ recovery}$, and E_i denotes the corresponding primary energy consumption or recovery which can be calculated following Supplementary Equations S16, S17, S18, and S15, respectively, in Supplementary Note 3; and ε is the CO_2 emission factor of coal equivalent, whose value can be found in Supplementary Table 4.

Supplementary Note 5

As is the case with calculating the manufacturing cost of other industrial products, cost of steel (COS, €/t) can be determined via Supplementary Equation S23 with several correlating techno-economic variables, including the annuity factor (α, yr^{-1}), the total capital requirement (I, €), the total annual cost of energy (C_{Energy}, €/yr), the total annual operation and maintenance (O&M) costs ($C_{\text{O\&M}}$, €/yr), the total annual cost of raw materials (C_{Material}, €/yr), and the total annual yield of steel products (M_{Steel}, t/yr).

$$COS = \frac{\alpha \cdot I + C_{\text{Energy}} + C_{\text{O\&M}} + C_{\text{Material}}}{M_{\text{Steel}}} \qquad \text{(S23)}$$

The annuity factor α can be calculated using the following equation:

$$\alpha = \frac{\theta \cdot (1+\theta)^n}{(1+\theta)^n - 1} \qquad \text{(S24)}$$

where θ and n denote the project interest rate (%) and economic lifetime (yr), respectively, whose valuation in this study can be found in Supplementary Table 4.

Supplementary Note 6

Limestone feed percent (f_p) is the basic variable for the operation of the CaL-LP scheme, and is prevalued within the typical range of 1-6% in this study. Here, detailed calculations revealing the integration of data, models, and methods involved in this study are illustrated as below at a limestone feed percent of 1% and 5%, respectively.

According to equation 4, if we set,

$$M_{CO_2}^{retrofit} = M_{CO_2}^{reference} + M_{CO_2}^{O_2\ separation} + M_{CO_2}^{CO_2\ compression} - M_{CO_2}^{lime\ kiln} - M_{CO_2}^{heat\ recovery} = \beta \cdot M_{CO_2}^{reference} \qquad (S25)$$

Then,

- ***<u>When f_p = 1%,</u>***

The maximum average carbonation conversion ($X_{ave,max}$) of lime in the add-on kiln is determined as 0.136 via equation 1 and Supplementary Equations S2 and S7. Therefore, the value of solid-looping ratio (R) and CO_2 capture efficiency ($\eta_{capt.}$) in the add-on kiln can be expressed as $\frac{7.333}{\beta}$ according to Supplementary Equation S9 and $\frac{0.997}{\beta}$ according to Supplementary Equation S8, respectively.

Now considering the energy and mass balance in the lime kiln of the CaL-LP scheme, terms $E_{cycled\ solid}$, $E_{fresh\ solid}$, E_{O_2}, and $E_{reaction}$ in equation 2 can be determined according to Supplementary Equations S11-S14, respectively, via the following calculations:

(I) $$E_{cycled\ solid} = \int_{T_{car.}}^{T_{cal.}} \frac{M_{limestone}}{MW_{CaCO_3} \cdot f_p} \cdot \left[X_{ave,max} \cdot CP_{CaCO_3}(T) + (1 - X_{ave,max}) \cdot CP_{CaO}(T) \right] \cdot dT$$

$$= \frac{M_{limestone}}{MW_{CaCO_3} \cdot f_p} \cdot \left\{ X_{ave,max} \cdot \left[A_1 \cdot (T_{cal.} - T_{car.}) + \frac{B_1 \cdot 10^{-3}}{2} \cdot (T_{cal.}{}^2 - T_{car.}{}^2) - C_1 \cdot 10^5 \cdot (\frac{1}{T_{cal.}} - \frac{1}{T_{car.}}) \right] + (1 - X_{ave,max}) \cdot \left[A_2 \cdot (T_{cal.} - T_{car.}) + \frac{B_2 \cdot 10^{-3}}{2} \cdot (T_{cal.}{}^2 - T_{car.}{}^2) - C_2 \cdot 10^5 \cdot (\frac{1}{T_{cal.}} - \frac{1}{T_{car.}}) \right] \right\}$$

$$= \frac{300\ kg/t_{steel}}{100\ kg/kmol \times 1\%} \times \left\{ \begin{aligned} &0.135958 \times \left[24.98 \times (1173.15 - 923.15) + 2.62 \times 10^{-3} \times (1173.15^2 - 923.15^2) + 6.20 \times 10^5 \times \left(\frac{1}{1173.15} - \frac{1}{923.15} \right) \right] \\ &+ (1 - 0.135958) \times \left[11.86 \times (1173.15 - 923.15) + 0.54 \times 10^{-3} \times (1173.15^2 - 923.15^2) + 1.66 \times 10^5 \times \left(\frac{1}{1173.15} - \frac{1}{923.15} \right) \right] \end{aligned} \right\}$$

$$= 1136.874\ Mcal/t_{steel} = 4758.953\ MJ/t_{steel}$$

(II) $$E_{fresh\ solid} = \int_{T_0}^{T_{cal.}} \frac{M_{limestone}}{MW_{CaCO_3}} \cdot CP_{CaCO_3}(T) \cdot dT$$

$$=\frac{M_{\text{limestone}}}{MW_{\text{CaCO}_3}}\cdot\left[A_1\cdot(T_{\text{cal.}}-T_0)+\frac{B_1\cdot10^{-3}}{2}\cdot(T_{\text{cal.}}{}^2-T_0{}^2)-C_1\cdot10^5\cdot(\frac{1}{T_{\text{cal.}}}-\frac{1}{T_0})\right]$$

$$=\frac{300\ \text{kg/t}_{\text{steel}}}{100\ \text{kg/kmol}}\times\left[24.98\times(1173.15-298.15)+2.62\times10^{-3}\times(1173.15^2-298.15^2)+6.20\times10^5\times\left(\frac{1}{1173.15}-\frac{1}{298.15}\right)\right]$$

$$=71.038\ \text{Mcal/t}_{\text{steel}}=297.367\ \text{MJ/t}_{\text{steel}}$$

(III) $$E_{\text{O}_2}=\int_{T_0}^{T_{\text{cal.}}}\frac{M_{\text{O}_2}}{MW_{\text{O}_2}}\cdot CP_{\text{O}_2}(T)\cdot\text{d}T$$

$$=\frac{M_{\text{O}_2}}{MW_{\text{O}_2}}\cdot\left[A_{\text{O}_2}\cdot(T_{\text{cal.}}-T_0)+\frac{B_{\text{O}_2}\cdot10^{-3}}{2}\cdot\left(T_{\text{cal.}}{}^2-T_0{}^2\right)-C_{\text{O}_2}\cdot10^5\cdot\left(\frac{1}{T_{\text{cal.}}}-\frac{1}{T_0}\right)\right]$$

$$=\frac{M_{\text{O}_2}}{32\ \text{kg/kmol}}\times\left[7.16\times(1173.15-298.15)+0.5\times10^{-3}\times(1173.15^2-298.15^2)+0.4\times10^5\times\left(\frac{1}{1173.15}-\frac{1}{298.15}\right)\right]$$

$$=212.770\cdot M_{\text{O}_2}\ \text{kcal/t}_{\text{steel}}=0.891\cdot M_{\text{O}_2}\ \text{MJ/t}_{\text{steel}}$$

(IV) $$E_{\text{reaction}}=\left(X_{\text{ave,max}}+f_{\text{p}}\right)\cdot\frac{M_{\text{limestone}}}{MW_{\text{CaCO}_3}\cdot f_{\text{p}}}\cdot\Delta H_{\text{cal.}}$$

$$=(0.135958+1\%)\times\frac{300\ \text{kg/t}_{\text{steel}}}{100\ \text{kg/kmol}\times1\%}\times166.042=7270.547\ \text{MJ/t}_{\text{steel}}$$

When the results in calculations (I)-(IV) are inserted into equation 2, the equation will be described as:

$$E_{\text{CO}_2\ \text{separation}}=4758.953+297.367+0.891\cdot M_{\text{O}_2}+7270.547$$

Upon introducing Supplementary Equation S10 and substituting the relationship of $M_{\text{O}_2}=FR_{\text{O}_2/\text{ce}}\cdot M_{\text{ce}}$ into the above equation, the fuel requirement in the lime kiln per tonne of crude steel produced is determined as 457.839 kg of coal equivalent, equaling the primary energy consumption of 13.415 GJ/$t_{\text{crude steel}}$, and resulting in O_2 consumption of 1.221 t/$t_{\text{crude steel}}$ and CO_2 generation of 1.163 t/$t_{\text{crude steel}}$. In addition, the total amount of CO_2 generated in the lime kiln and required for compression can be calculated as follows:

$$[M]_{\text{CO}_2}^{\text{compression}}=[M]_{\text{CO}_2}^{\text{separation}}+[M]_{\text{CO}_2}^{\text{limestone}}+[M]_{\text{CO}_2}^{\text{combustion}}$$

$$=\eta_{\text{capt.}}\cdot M_{\text{CO}_2}^{\text{retrofit}}+\frac{MW_{\text{CO}_2}}{MW_{\text{CaCO}_3}}\cdot M_{\text{limestone}}+2.54\cdot M_{\text{ce}}=\frac{0.997}{\beta}\times1.80\times\beta+\frac{44}{100}\times0.3+2.54\times0.457839=3.089\ \text{t/t}_{\text{steel}}$$

Since the amount of O_2 required and CO_2 ready for compression during the operation of the CaL-LP scheme have been determined, the incremental primary energy consumption for O_2 separation and CO_2 compression is 1.889 GJ/$t_{\text{crude steel}}$ (64.474 kg_{ce}/$t_{\text{crude steel}}$) according to Supplementary Equation S16 and 2.867 GJ/$t_{\text{crude steel}}$ (97.865

$kg_{ce}/t_{crude\ steel}$) according to Supplementary Equation S17, respectively, resulting in a corresponding CO_2 generation of 163.764 $kg/t_{crude\ steel}$ and 248.578 $kg/t_{crude\ steel}$.

Now turning to the conventional lime kiln, the energy balance can be described using Supplementary Equation S18, where $E_{\text{limestone}}$, E_{air}, and $E_{\text{decomposition}}$ can be determined according to Supplementary Equations S19-S21, respectively, via the following calculations:

$$\text{(I)}\ E_{\text{limestone}} = \int_{T_0}^{T_{\text{cal.}}} \frac{M_{\text{limestone}}}{MW_{\text{CaCO}_3}} \cdot CP_{\text{CaCO}_3}(T) \cdot \text{d}T$$

$$= \frac{M_{\text{limestone}}}{MW_{\text{CaCO}_3}} \cdot \left[A\cdot(T_{\text{cal.}} - T_0) + \frac{B\cdot 10^{-3}}{2}\cdot(T_{\text{cal.}}{}^2 - T_0^2) - C\cdot 10^5 \cdot (\frac{1}{T_{\text{cal.}}} - \frac{1}{T_0}) + \frac{D\cdot 10^{-6}}{3}\cdot(T_{\text{cal.}}{}^3 - T_0^3) \right]$$

$$= \frac{300\ \text{kg/t}_{\text{steel}}}{100\ \text{kg/kmol}} \times \left[24.98\times(1173.15-298.15) + 2.62\times10^{-3}\times(1173.15^2 - 298.15^2) + 6.20\times10^5\times(\frac{1}{1173.15} - \frac{1}{298.15}) \right]$$

$$= 71.04\ \text{Mcal/t}_{\text{steel}} = 297.37\ \text{MJ/t}_{\text{steel}}$$

$$\text{(II)}\ E_{\text{air}} = \int_{T_0}^{T_{\text{cal.}}} [\frac{M_{\text{O}_2}}{MW_{\text{O}_2}} \cdot CP_{\text{O}_2}(T) + \frac{78\%}{21\%} \cdot \frac{M_{\text{O}_2}}{MW_{\text{O}_2}} \cdot CP_{\text{N}_2}(T)] \cdot \text{d}T$$

$$= \frac{M_{\text{O}_2}}{MW_{\text{O}_2}} \cdot \left[(A_{\text{O}_2} + \frac{78}{21}\times A_{\text{N}_2})\cdot(T_{\text{cal.}} - T_0) + \frac{10^{-3}}{2}\cdot(B_{\text{O}_2} + \frac{78}{21}\times B_{\text{N}_2})\cdot(T_{\text{cal.}}{}^2 - T_0^2) - 10^5\cdot(C_{\text{O}_2} + \frac{78}{21}\times C_{\text{N}_2})\cdot(\frac{1}{T_{\text{cal.}}} - \frac{1}{T_0}) \right]$$

$$= \frac{M_{\text{O}_2}}{32\ \text{kg/kmol}} \times \left[(7.16 + \frac{78}{21}\times 6.66)\times(1173.15-298.15) + 10^{-3}\times(0.5 + \frac{78}{21}\times 0.51)\times(1173.15^2 - 298.15^2) + 0.4\times10^5\times(\frac{1}{1173.15} - \frac{1}{298.15}) \right]$$

$$= 965.38\cdot M_{\text{O}_2}\ \text{kcal/t}_{\text{steel}} = 4.04\cdot M_{\text{O}_2}\ \text{MJ/t}_{\text{steel}}$$

$$\text{(III)}\ E_{\text{decomposition}} = \frac{M_{\text{limestone}}}{MW_{\text{CaCO}_3}} \cdot \Delta H_{\text{cal.}}$$

$$= \frac{300\ \text{kg/t}_{\text{steel}}}{100\ \text{kg/kmol}} \times 166.042\ \text{kJ/mol} = 498.13\ \text{MJ/t}_{\text{steel}}$$

When the results in calculations (I)-(III) are inserted into Supplementary Equation S18, the equation will be described as:

$$E_{\text{lime kiln}} = 297.37 + 4.04\cdot M_{\text{O}_2} + 498.13$$

Upon introducing Supplementary Equation S10 and substituting the relationship of $M_{\text{O}_2} = FR_{\text{O}_2/\text{ce}} \cdot M_{\text{ce}}$ into the above equation, the fuel requirement in the conventional lime kiln per tonne of crude steel produced is determined as 42.938 kg of coal equivalent, equaling the primary energy consumption of 1.258 $GJ/t_{crude\ steel}$, and resulting in the CO_2 emission of 241.063 $kg/t_{crude\ steel}$ when the process CO_2 emission of 132.000 $kg/t_{crude\ steel}$ due to the limestone decomposition reaction is considered.

Due to the implementation of heat integration measures (commonly considered during the application of calcium-looping technology), 836.072 kWh/$t_{crude\ steel}$ of electricity can be recovered theoretically from the add-on kiln of the CaL-LP scheme according to Supplementary Equation S15. This will save a fuel consumption of 238.897 $kg_{ce}/t_{crude\ steel}$, equaling a reduction in CO_2 generation of 606.798 kg/$t_{crude\ steel}$.

Finally, when we substitute $M_{CO_2}^{O_2\ \text{separation}} = 163.764$ kg/$t_{crude\ steel}$, $M_{CO_2}^{CO_2\ \text{compression}} = 248.578$ kg/$t_{crude\ steel}$, $M_{CO_2}^{\text{lime kiln}} = 241.063$ kg/$t_{crude\ steel}$, and $M_{CO_2}^{\text{heat recovery}} = 606.798$ kg/$t_{crude\ steel}$ into Supplementary Equation S25, the value of β will be determined, and it is 0.758 here when f_p=1%.

However, when β=0.758, $\eta_{\text{capt.}} = \frac{0.997}{\beta} = \frac{0.997}{0.758} = 1.315 > 1$, indicating that the realistic conversion of lime ($X_{\text{real.}}$) in the add-on kiln does not arrive at the maximum average carbonation conversion of $X_{\text{ave,max}} = 0.136$, while the CO_2 capture efficiency $\eta_{\text{capt.}}$ has already reached 100%. Therefore, we can express the value of $X_{\text{real.}}$ as $\frac{\beta}{7.333}$ according to Supplementary Equation S8.

Reconsidering the energy and mass balance in the lime kiln of the CaL-LP scheme,

$$E_{\text{cycled solid}} = \int_{T_{\text{car.}}}^{T_{\text{cal.}}} \frac{M_{\text{limestone}}}{MW_{CaCO_3} \cdot f_{\text{p}}} \cdot \left[X_{\text{real.}} \cdot CP_{CaCO_3}(T) + (1 - X_{\text{real.}}) \cdot CP_{CaO}(T) \right] \cdot \mathrm{d}T$$

$$= \frac{M_{\text{limestone}}}{MW_{CaCO_3} \cdot f_{\text{p}}} \cdot \left\{ X_{\text{real.}} \cdot \left[A_1 \cdot (T_{\text{cal.}} - T_{\text{car.}}) + \frac{B_1 \cdot 10^{-3}}{2} \cdot (T_{\text{cal.}}{}^2 - T_{\text{car.}}{}^2) - C_1 \cdot 10^5 \cdot (\frac{1}{T_{\text{cal.}}} - \frac{1}{T_{\text{car.}}}) \right] + (1 - X_{\text{real.}}) \cdot \left[A_2 \cdot (T_{\text{cal.}} - T_{\text{car.}}) + \frac{B_2 \cdot 10^{-3}}{2} \cdot (T_{\text{cal.}}{}^2 - T_{\text{car.}}{}^2) - C_2 \cdot 10^5 \cdot (\frac{1}{T_{\text{cal.}}} - \frac{1}{T_{\text{car.}}}) \right] \right\}$$

$$= \frac{300\ \text{kg/t}_{\text{steel}}}{100\ \text{kg/kmol} \times 1\%} \times \left\{ \begin{array}{l} \frac{\beta}{7.333} \times \left[24.98 \times (1173.15 - 923.15) + 2.62 \times 10^{-3} \times (1173.15^2 - 923.15^2) + 6.20 \times 10^5 \times \left(\frac{1}{1173.15} - \frac{1}{923.15} \right) \right] \\ + \left(1 - \frac{\beta}{7.333} \right) \times \left[11.86 \times (1173.15 - 923.15) + 0.54 \times 10^{-3} \times (1173.15^2 - 923.15^2) + 1.66 \times 10^5 \times \left(\frac{1}{1173.15} - \frac{1}{923.15} \right) \right] \end{array} \right\}$$

$$= (962.904 + 174.488 \cdot \beta)\ \text{Mcal/t}_{\text{steel}} = (4030.717 + 730.409 \cdot \beta)\ \text{MJ/t}_{\text{steel}}$$

$$E_{\text{reaction}} = \left(X_{\text{real.}} + f_{\text{p}} \right) \cdot \frac{M_{\text{limestone}}}{MW_{CaCO_3} \cdot f_{\text{p}}} \cdot \Delta H_{\text{cal.}}$$

$$= \left(\frac{\beta}{7.333} + 1\% \right) \times \frac{300\ \text{kg/t}_{\text{steel}}}{100\ \text{kg/kmol} \times 1\%} \times 166.042 = (498.126 + 6792.627 \cdot \beta)\ \text{MJ/t}_{\text{steel}}$$

while the calculations of $E_{\text{fresh solid}}$ and E_{O_2} remain unchanged. According to equation 2,

$$E_{CO_2\ separation} = (4030.717 + 730.409 \cdot \beta) + 297.367 + 0.891 \cdot M_{O_2} + (498.126 + 6792.627 \cdot \beta)$$

Upon introducing Supplementary Equation S10 and substituting the relationship of $M_{O_2} = FR_{O_2/ce} \cdot M_{ce}$ into the above equation, the fuel requirement in the lime kiln per tonne of crude steel produced is determined as $(179.253 + 279.417 \cdot \beta)$ kg of coal equivalent, equaling the primary energy consumption of $(5.252 + 8.187 \cdot \beta)$ GJ/$t_{crude\ steel}$, and resulting in the O_2 consumption of $(0.478 + 0.745 \cdot \beta)$ t/$t_{crude\ steel}$ and CO_2 generation of $(0.455 + 0.710 \cdot \beta)$ t/$t_{crude\ steel}$.

In addition, the total amount of CO_2 generated in the lime kiln and required for compression can be calculated as follows:

$$[M]_{CO_2}^{compression} == \eta_{capt.} \cdot M_{CO_2}^{retrofit} + \frac{MW_{CO_2}}{MW_{CaCO_3}} \cdot M_{limestone} + 2.54 \cdot M_{ce}$$

$$= 1 \times 1.80 \times \beta + \frac{44}{100} \times 0.3 + 2.54 \times \frac{(179.253 + 279.417 \cdot \beta)}{1000} = (0.587 + 2.510 \cdot \beta) \text{ t/t}_{steel}$$

Therefore, the incremental primary energy consumption for O_2 separation and CO_2 compression is $(0.739 + 1.153 \cdot \beta)$ GJ/$t_{crude\ steel}$ ($(25.240 + 39.339 \cdot \beta)$ kg_{ce}/$t_{crude\ steel}$) according to Supplementary Equation S16 and $(0.545 + 2.330 \cdot \beta)$ GJ/$t_{crude\ steel}$ ($(18.597 + 79.521 \cdot \beta)$ kg_{ce}/$t_{crude\ steel}$) according to Supplementary Equation S17, respectively, resulting in a corresponding CO_2 generation of $(64.111 + 99.921 \cdot \beta)$ kg/$t_{crude\ steel}$ and $(47.237 + 201.985 \cdot \beta)$ kg/$t_{crude\ steel}$.

According to Supplementary Equation S15, $838.588 \cdot \beta$ kWh/$t_{crude\ steel}$ of electricity can be recovered theoretically from the add-on kiln of the CaL-LP scheme, saving fuel consumption of $239.616 \cdot \beta$ kg_{ce}/$t_{crude\ steel}$, equaling a reduction in CO_2 generation of $608.624 \cdot \beta$ kg/$t_{crude\ steel}$.

Again, when we substitute $M_{CO_2}^{O_2\ separation} = (64.111 + 99.921 \cdot \beta)$ kg/$t_{crude\ steel}$, $M_{CO_2}^{CO_2\ compression} = (47.237 + 201.985 \cdot \beta)$ kg/$t_{crude\ steel}$, $M_{CO_2}^{lime\ kiln} = 241.063$ kg/$t_{crude\ steel}$,

and $M_{CO_2}^{\text{heat recovery}} = 608.624 \cdot \beta$ kg/t$_{\text{crude steel}}$ into Supplementary Equation S25, the value of β is determined to be 0.793; therefore, $M_{CO_2}^{\text{retrofit}} = 1427.058$ kg/t$_{\text{crude steel}}$ and R=9.248. Overall, the techno-economic performance of the conventional integrated steel mill retrofitted with the CaL-LP scheme when f_p=1% can be summarised as:

Energy consumption:

1) the incremental primary energy consumption per tonne of crude steel produced:

$$\begin{aligned}\Delta E_{\text{crude steel}} &= E_{CO_2\ \text{separation}} + E_{O_2\ \text{separation}} + E_{CO_2\ \text{compression}} - E_{\text{lime kiln}} - E_{\text{heat recovery}} \\ &= (5.252 + 8.187 \cdot \beta) + (0.739 + 1.153 \cdot \beta) + (0.545 + 2.330 \cdot \beta) - 1.258 - 29.3 \times 239.616 \cdot \beta \div 1000 \\ &= 8.965\ \text{GJ/t}_{\text{crude steel}}\end{aligned}$$

When we further break this incremental energy consumption $\Delta E_{\text{crude steel}}$ down,

- $E_{CO_2\ \text{separation}} = 5.252 + 8.187 \times 0.793 = 11.744$ GJ/t$_{\text{crude steel}}$, accounting for 131.0% of the total incremental energy consumption $\Delta E_{\text{crude steel}}$;
- $E_{O_2\ \text{separation}} = 0.739 + 1.153 \times 0.793 = 1.653$ GJ/t$_{\text{crude steel}}$, accounting for 18.4% of $\Delta E_{\text{crude steel}}$;
- $E_{CO_2\ \text{compression}} = 0.545 + 2.330 \times 0.793 = 2.393$ GJ/t$_{\text{crude steel}}$, accounting for 26.7% of $\Delta E_{\text{crude steel}}$;
- $E_{\text{lime kiln}} = -1.258$ GJ/t$_{\text{crude steel}}$, accounting for -14.0% of $\Delta E_{\text{crude steel}}$;
- $E_{\text{heat recovery}} = -29.3 \times 239.616 \times 0.793 \div 1000 = -5.567$ GJ/t$_{\text{crude steel}}$, accounting for -62.1% of $\Delta E_{\text{crude steel}}$.

2) the primary energy consumption per tonne of CO_2 reduced:

$$\begin{aligned}E_{CO_2} &= \frac{\Delta E_{\text{crude steel}}}{(EOC)_{\text{reference}} - (EOC)_{\text{retrofit}}} = \frac{E_{CO_2\ \text{separation}} + E_{O_2\ \text{separation}} + E_{CO_2\ \text{compression}} - E_{\text{lime kiln}} - E_{\text{heat recovery}}}{M_{CO_2}^{\text{reference}} - (1 - \eta_{\text{capt.}}) \cdot M_{CO_2}^{\text{retrofit}}} \\ &= \frac{8.965}{1.800 - 1.427 \times (1-1)} = 4.980\ \text{GJ/t}_{CO_2}\end{aligned}$$

When we further break this energy consumption, E_{CO_2}, down,

- $$E'_{CO_2\ separation}=\frac{E_{CO_2\ separation}}{M_{CO_2}^{reference}-\left(1-\eta_{capt.}\right)\cdot M_{CO_2}^{retrofit}}=\frac{E_{CO_2\ separation}}{M_{CO_2}^{reference}}=\frac{11.744}{1.8}=6.524\ \mathrm{GJ/t_{CO_2}}$$,

  accounting for 131.0% of the total energy consumption E_{CO_2};

- $$E'_{O_2\ separation}=\frac{E_{O_2\ separation}}{M_{CO_2}^{reference}-\left(1-\eta_{capt.}\right)\cdot M_{CO_2}^{retrofit}}=\frac{E_{O_2\ separation}}{M_{CO_2}^{reference}}=\frac{1.653}{1.8}=0.918\ \mathrm{GJ/t_{CO_2}}$$,

  accounting for 18.4% of E_{CO_2};

- $$E'_{CO_2\ compression}=\frac{E_{CO_2\ compression}}{M_{CO_2}^{reference}-\left(1-\eta_{capt.}\right)\cdot M_{CO_2}^{retrofit}}=\frac{E_{CO_2\ compression}}{M_{CO_2}^{reference}}=\frac{2.393}{1.8}=1.329\ \mathrm{GJ/t_{CO_2}}$$,

  accounting for 26.7% of E_{CO_2};

- $$E'_{lime\ kiln}=\frac{E_{lime\ kiln}}{M_{CO_2}^{reference}-\left(1-\eta_{capt.}\right)\cdot M_{CO_2}^{retrofit}}=\frac{E_{lime\ kiln}}{M_{CO_2}^{reference}}=\frac{-1.258}{1.8}=-0.699\ \mathrm{GJ/t_{CO_2}}$$,

  accounting for -14.0% of E_{CO_2};

- $$E'_{heat\ recovery}=\frac{E_{heat\ recovery}}{M_{CO_2}^{reference}-\left(1-\eta_{capt.}\right)\cdot M_{CO_2}^{retrofit}}=\frac{E_{heat\ recovery}}{M_{CO_2}^{reference}}=\frac{-5.567}{1.8}=-3.093\ \mathrm{GJ/t_{CO_2}}$$,

  accounting for -62.1% of E_{CO_2}.

<u>CO_2 reduction:</u>

<u>1) the total amount of CO_2 reduced per tonne of crude steel produced:</u>

$$\Delta M_{CO_2}=M_{CO_2}^{reference}-\left(1-\eta_{capt.}\right)\cdot M_{CO_2}^{retrofit}=1.800-1.427\times\left(1-1\right)=1.800\ \mathrm{t_{CO_2}/t_{crude\ steel}}$$

When we further break this amount of CO_2 reduced, ΔM_{CO_2} , down,

- Amount of CO_2 reduced from the add-on kiln of the CaL-LP scheme:

  $$\Delta M_{CO_2}^{add\text{-}on\ kiln}=\eta_{capt.}\cdot M_{CO_2}^{retrofit}=100\%\times1.427=1.427\ \mathrm{t_{CO_2}/t_{crude\ steel}}$$

- Amount of CO_2 reduced from the lime kiln of the CaL-LP scheme:

  $$\Delta M_{CO_2}^{lime\ kiln}=\frac{MW_{CO_2}}{MW_{CaCO_3}}\cdot M_{limestone}+\frac{2.54\times E_{lime\ kiln}}{29.3}=\frac{44}{100}\times0.3+\frac{2.54\times1.258}{29.3}=0.241\ \mathrm{t_{CO_2}/t_{crude\ steel}}$$

- Amount of CO_2 reduced due to the net electricity recovered from the CaL-LP scheme:

  $$\Delta M_{CO_2}^{electricity}=M_{CO_2}^{heat\ recovery}-M_{CO_2}^{O_2\ separation}-M_{CO_2}^{CO_2\ compression}$$
  $$=0.609\cdot\beta-\left(0.064+0.100\cdot\beta\right)-\left(0.047+0.202\cdot\beta\right)=0.307\cdot\beta-0.111=0.132\ \mathrm{t_{CO_2}/t_{crude\ steel}}$$

2) overall CO_2 reduction efficiency:

$$\varphi_{CO_2} = \frac{M_{CO_2}^{\text{reference}} - \left(1-\eta_{\text{capt.}}\right)\cdot M_{CO_2}^{\text{retrofit}}}{M_{CO_2}^{\text{reference}}}\times 100\% = \frac{1.800 - 1.427\times\left(1-1\right)}{1.800}\times 100\% = 100\%$$

When we further break the overall CO_2 reduction efficiency φ_{CO_2} down,

- CO_2 reduction efficiency from the add-on kiln of the CaL-LP scheme:

$$\varphi_{CO_2}^{\text{add-on kiln}} = \frac{\Delta M_{CO_2}^{\text{add-on kiln}}}{\Delta M_{CO_2}}\times 100\% = \frac{1.427}{1.800}\times 100\% = 79.3\%$$

- CO_2 reduction efficiency from the lime kiln of the CaL-LP scheme:

$$\varphi_{CO_2}^{\text{lime kiln}} = \frac{\Delta M_{CO_2}^{\text{lime kiln}}}{\Delta M_{CO_2}}\times 100\% = \frac{0.241}{1.800}\times 100\% = 13.4\%$$

- CO_2 reduction efficiency due to the net electricity recovered from the CaL-LP scheme: $\varphi_{CO_2}^{\text{electricity}} = \frac{\Delta M_{CO_2}^{\text{electricity}}}{\Delta M_{CO_2}}\times 100\% = \frac{0.132}{1.800}\times 100\% = 7.3\%$

Cost of steel production and CO_2 reduction:

1) the incremental cost of crude steel:

$$\Delta C_{\text{crude steel}} = \left(COS\right)_{\text{retrofit}} - \left(COS\right)_{\text{reference}} = \alpha\cdot\Delta I' + \Delta C'_{\text{Energy}} + \Delta C'_{\text{O\&M}} + \Delta C'_{\text{Material}}$$
$$= 0.11\times\left(45+25\right) + 1.5\times 8.965 + 4\%\times\left(45+25\right) + 0 = 23.95\ €_{2010}/\text{t}_{\text{crude steel}}$$

When we further break the incremental cost of crude steel, $\Delta C_{\text{crude steel}}$, down,

- Cost contribution from the incremental capital requirement due to implementation of the CaL-LP scheme:

$$\Delta C_{\text{crude steel}}^{\text{capital}} = \alpha\cdot\Delta I' = 0.11\times\left(45+25\right) = 7.70\ €_{2010}/\text{t}_{\text{crude steel}}$$
$$\omega_{\text{capital}} = \frac{\Delta C_{\text{crude steel}}^{\text{capital}}}{\Delta C_{\text{crude steel}}}\times 100\% = \frac{7.70}{23.95}\times 100\% = 32.1\%$$

- Cost contribution from the incremental cost of energy due to implementation of the CaL-LP scheme:

$$\Delta C_{\text{crude steel}}^{\text{energy}} = \Delta C'_{\text{Energy}} = EP\cdot\Delta E_{\text{crude steel}} = 1.5\times 8.965 = 13.45\ €_{2010}/\text{t}_{\text{crude steel}}$$
$$\omega_{\text{energy}} = \frac{\Delta C_{\text{crude steel}}^{\text{energy}}}{\Delta C_{\text{crude steel}}}\times 100\% = \frac{13.45}{23.95}\times 100\% = 56.2\%$$

- Cost contribution from the incremental operation and maintenance (O&M) costs due to implementation of the CaL-LP scheme:

$$\Delta C^{\text{O\&M}}_{\text{crude steel}} = \Delta C'_{\text{O\&M}} = \mu \cdot \Delta I' = 4\% \times (45+25) = 2.80\ €_{2010}/\text{t}_{\text{crude steel}}$$

$$\omega_{\text{O\&M}} = \frac{\Delta C^{\text{O\&M}}_{\text{crude steel}}}{\Delta C_{\text{crude steel}}} \times 100\% = \frac{2.80}{23.95} \times 100\% = 11.7\%$$

- Cost contribution from the incremental cost of raw materials due to implementation of the CaL-LP scheme is almost zero, since no additional material consumption is required.

2) CO_2 avoidance cost:

$$AC = \frac{\alpha \cdot \Delta I' + \Delta C'_{\text{Energy}} + \Delta C'_{\text{O\&M}} + \Delta C'_{\text{Material}}}{(EOC)_{\text{reference}} - (EOC)_{\text{retrofit}}} = \frac{\Delta C_{\text{crude steel}}}{\Delta M_{\text{CO}_2}} = \frac{23.95\ €_{2010}/\text{t}_{\text{crude steel}}}{1.800\ \text{t}_{\text{CO}_2}/\text{t}_{\text{crude steel}}} = 13.30\ €_{2010}/\text{t}_{\text{CO}_2}$$

When we further break the CO_2 avoidance cost, AC, down,

- Cost contribution from the incremental capital requirement due to implementation of the CaL-LP scheme:

$$AC_{\text{capital}} = \frac{\alpha \cdot \Delta I'}{\Delta M_{\text{CO}_2}} = \frac{0.11 \times (45+25)}{1.800} = 4.28\ €_{2010}/\text{t}_{\text{CO}_2}$$

$$\varpi_{\text{capital}} = \frac{AC_{\text{capital}}}{AC} \times 100\% = \frac{4.28}{13.30} \times 100\% = 32.1\%$$

- Cost contribution from the incremental cost of energy due to implementation of the CaL-LP scheme:

$$AC_{\text{energy}} = \frac{EP \cdot \Delta E_{\text{crude steel}}}{\Delta M_{\text{CO}_2}} = \frac{1.5 \times 8.965}{1.800} = 7.47\ €_{2010}/\text{t}_{\text{CO}_2}$$

$$\varpi_{\text{energy}} = \frac{AC_{\text{energy}}}{AC} \times 100\% = \frac{7.47}{13.30} \times 100\% = 56.2\%$$

- Cost contribution from the incremental operation and maintenance (O&M) costs due to implementation of the CaL-LP scheme:

$$AC_{\text{O\&M}} = \frac{\mu \cdot \Delta I'}{\Delta M_{\text{CO}_2}} = \frac{4\% \times (45+25)}{1.800} = 1.55\ €_{2010}/\text{t}_{\text{CO}_2}$$

$$\omega_{\text{O\&M}} = \frac{AC_{\text{O\&M}}}{AC} \times 100\% = \frac{1.55}{13.30} \times 100\% = 11.7\%$$

- Cost contribution from the incremental cost of raw materials due to implementation of the CaL-LP scheme is almost zero, since no additional material consumption is required.

When $f_p = 5\%$,

$X_{\text{ave,max}}$ of lime in the add-on kiln is determined as 0.246, and the values of R and $\eta_{\text{capt.}}$ will become $\dfrac{1.467}{\beta}$ and $\dfrac{0.362}{\beta}$, respectively.

In the lime kiln,

$$\text{(I)}\quad E_{\text{cycled solid}} = \int_{T_{\text{car.}}}^{T_{\text{cal.}}} \frac{M_{\text{limestone}}}{MW_{\text{CaCO}_3} \cdot f_{\text{p}}} \cdot \left[X_{\text{ave,max}} \cdot CP_{\text{CaCO}_3}\left(T\right) + \left(1 - X_{\text{ave,max}}\right) \cdot CP_{\text{CaO}}\left(T\right) \right] \cdot \mathrm{d}T$$

$$= \frac{M_{\text{limestone}}}{MW_{\text{CaCO}_3} \cdot f_{\text{p}}} \cdot \left\{ X_{\text{ave,max}} \cdot \left[A_1 \cdot (T_{\text{cal.}} - T_{\text{car.}}) + \frac{B_1 \cdot 10^{-3}}{2} \cdot (T_{\text{cal.}}{}^2 - T_{\text{car.}}{}^2) - C_1 \cdot 10^5 \cdot (\frac{1}{T_{\text{cal.}}} - \frac{1}{T_{\text{car.}}}) \right] + (1 - X_{\text{ave,max}}) \cdot \left[A_2 \cdot (T_{\text{cal.}} - T_{\text{car.}}) + \frac{B_2 \cdot 10^{-3}}{2} \cdot (T_{\text{cal.}}{}^2 - T_{\text{car.}}{}^2) - C_2 \cdot 10^5 \cdot (\frac{1}{T_{\text{cal.}}} - \frac{1}{T_{\text{car.}}}) \right] \right\}$$

$$= \frac{300\ \text{kg/t}_{\text{steel}}}{100\ \text{kg/kmol} \times 5\%} \times \left\{ \begin{aligned} & 0.246497 \times \left[24.98 \times (1173.15 - 923.15) + 2.62 \times 10^{-3} \times (1173.15^2 - 923.15^2) + 6.20 \times 10^5 \times \left(\frac{1}{1173.15} - \frac{1}{923.15} \right) \right] \\ & + (1 - 0.246497) \times \left[11.86 \times (1173.15 - 923.15) + 0.54 \times 10^{-3} \times (1173.15^2 - 923.15^2) + 1.66 \times 10^5 \times \left(\frac{1}{1173.15} - \frac{1}{923.15} \right) \right] \end{aligned} \right\}$$

$$= 255.663\ \text{Mcal/t}_{\text{steel}} = 1070.207\ \text{MJ/t}_{\text{steel}}$$

$$\text{(II)}\quad E_{\text{fresh solid}} = \int_{T_0}^{T_{\text{cal.}}} \frac{M_{\text{limestone}}}{MW_{\text{CaCO}_3}} \cdot CP_{\text{CaCO}_3}\left(T\right) \cdot \mathrm{d}T$$

$$= \frac{M_{\text{limestone}}}{MW_{\text{CaCO}_3}} \cdot \left[A_1 \cdot (T_{\text{cal.}} - T_0) + \frac{B_1 \cdot 10^{-3}}{2} \cdot (T_{\text{cal.}}{}^2 - T_0^2) - C_1 \cdot 10^5 \cdot (\frac{1}{T_{\text{cal.}}} - \frac{1}{T_0}) \right]$$

$$= \frac{300\ \text{kg/t}_{\text{steel}}}{100\ \text{kg/kmol}} \times \left[24.98 \times (1173.15 - 298.15) + 2.62 \times 10^{-3} \times (1173.15^2 - 298.15^2) + 6.20 \times 10^5 \times \left(\frac{1}{1173.15} - \frac{1}{298.15} \right) \right]$$

$$= 71.038\ \text{Mcal/t}_{\text{steel}} = 297.367\ \text{MJ/t}_{\text{steel}}$$

$$\text{(III)}\quad E_{\text{O}_2} = \int_{T_0}^{T_{\text{cal.}}} \frac{M_{\text{O}_2}}{MW_{\text{O}_2}} \cdot CP_{\text{O}_2}\left(T\right) \cdot \mathrm{d}T$$

$$= \frac{M_{\text{O}_2}}{MW_{\text{O}_2}} \cdot \left[A_{\text{O}_2} \cdot (T_{\text{cal.}} - T_0) + \frac{B_{\text{O}_2} \cdot 10^{-3}}{2} \cdot (T_{\text{cal.}}{}^2 - T_0^2) - C_{\text{O}_2} \cdot 10^5 \cdot \left(\frac{1}{T_{\text{cal.}}} - \frac{1}{T_0} \right) \right]$$

$$= \frac{M_{\text{O}_2}}{32\ \text{kg/kmol}} \times \left[7.16 \times (1173.15 - 298.15) + 0.5 \times 10^{-3} \times (1173.15^2 - 298.15^2) + 0.4 \times 10^5 \times \left(\frac{1}{1173.15} - \frac{1}{298.15} \right) \right]$$

$$= 212.770 \cdot M_{\text{O}_2}\ \text{kcal/t}_{\text{steel}} = 0.891 \cdot M_{\text{O}_2}\ \text{MJ/t}_{\text{steel}}$$

$$\text{(IV)}\quad E_{\text{reaction}} = \left(X_{\text{ave,max}} + f_{\text{p}} \right) \cdot \frac{M_{\text{limestone}}}{MW_{\text{CaCO}_3} \cdot f_{\text{p}}} \cdot \Delta H_{\text{cal.}}$$

$$= (0.246497 + 5\%) \times \frac{300\ \text{kg/t}_{\text{steel}}}{100\ \text{kg/kmol} \times 5\%} \times 166.042 = 2953.857\ \text{MJ/t}_{\text{steel}}$$

When the results in calculations (I)~(IV) are inserted into equation 2, the equation will be described as:

$$E_{\text{CO}_2\ \text{separation}} = 1070.207 + 297.367 + 0.891 \cdot M_{\text{O}_2} + 2953.857$$

Upon introducing Supplementary Equation S10 and substituting the relationship of

$M_{O_2} = FR_{O_2/ce} \cdot M_{ce}$ into the above equation, the fuel requirement in the lime kiln per tonne of crude steel produced is determined as 160.505 kg of coal equivalent, equaling the primary energy consumption of 4.703 GJ/$t_{crude\ steel}$, and resulting in O_2 consumption of 428.013 kg/$t_{crude\ steel}$ and CO_2 generation of 407.682 kg/$t_{crude\ steel}$. In addition, the total amount of CO_2 generated in the lime kiln and required for compression can be calculated as follows:

$$[M]_{CO_2}^{compression} = [M]_{CO_2}^{separation} + [M]_{CO_2}^{limestone} + [M]_{CO_2}^{combustion}$$

$$= \eta_{capt.} \cdot M_{CO_2}^{retrofit} + \frac{MW_{CO_2}}{MW_{CaCO_3}} \cdot M_{limestone} + 2.54 \cdot M_{ce} = \frac{0.362}{\beta} \times 1.80 \times \beta + \frac{44}{100} \times 0.3 + 2.54 \times 0.160505 = 1.191 \text{ t/t}_{steel}$$

Therefore, the incremental primary energy consumption for O_2 separation and CO_2 compression is 0.662 GJ/$t_{crude\ steel}$ (22.601 kg_{ce}/$t_{crude\ steel}$) according to Supplementary Equation S16 and 1.106 GJ/$t_{crude\ steel}$ (37.733 kg_{ce}/$t_{crude\ steel}$) according to Supplementary Equation S17, respectively, resulting in a corresponding CO_2 generation of 57.406 kg/$t_{crude\ steel}$ and 95.842 kg/$t_{crude\ steel}$.

In the conventional lime kiln, the fuel requirement per tonne of crude steel produced is still 42.938 kg of coal equivalent, equaling the primary energy consumption of 1.258 GJ/$t_{crude\ steel}$, and resulting in the CO_2 emission of 241.063 kg/$t_{crude\ steel}$.

According to Supplementary Equation S15, 303.569 kWh/$t_{crude\ steel}$ of electricity can be recovered theoretically from the add-on kiln of the CaL-LP scheme. This will save fuel consumption of 86.741 kg_{ce}/$t_{crude\ steel}$, equaling a reduction in CO_2 generation of 220.322 kg/$t_{crude\ steel}$.

Finally, when we substitute $M_{CO_2}^{O_2\ separation} = 57.406$ kg/$t_{crude\ steel}$, $M_{CO_2}^{CO_2\ compression} = 95.842$ kg/$t_{crude\ steel}$, $M_{CO_2}^{lime\ kiln} = 241.063$ kg/$t_{crude\ steel}$, and $M_{CO_2}^{heat\ recovery} = 220.322$ kg/$t_{crude\ steel}$ into Supplementary Equation S25, the value of β is determined to be 0.829; therefore, $M_{CO_2}^{retrofit} = 1491.863$ kg/$t_{crude\ steel}$ and R=1.770.

Overall, the techno-economic performance of the conventional integrated steel mill retrofitted with the CaL-LP scheme when f_p=5% can be summarised as:

Energy consumption:

1) the incremental primary energy consumption per tonne of crude steel produced:

$$\Delta E_{\text{crude steel}} = E_{CO_2\ \text{separation}} + E_{O_2\ \text{separation}} + E_{CO_2\ \text{compression}} - E_{\text{lime kiln}} - E_{\text{heat recovery}}$$
$$= 4.703 + 0.662 + 1.106 - 1.258 - 29.3 \times 86.741 \div 1000 = 2.671\ \text{GJ/t}_{\text{crude steel}}$$

When we further break this incremental energy consumption, $\Delta E_{\text{crude steel}}$, down,

- $E_{CO_2\ \text{separation}} = 4.703\ \text{GJ/t}_{\text{crude steel}}$, accounting for 176.1% of the total incremental energy consumption $\Delta E_{\text{crude steel}}$;
- $E_{O_2\ \text{separation}} = 0.662\ \text{GJ/t}_{\text{crude steel}}$, accounting for 24.8% of $\Delta E_{\text{crude steel}}$;
- $E_{CO_2\ \text{compression}} = 1.106\ \text{GJ/t}_{\text{crude steel}}$, accounting for 41.4% of $\Delta E_{\text{crude steel}}$;
- $E_{\text{lime kiln}} = -1.258\ \text{GJ/t}_{\text{crude steel}}$, accounting for -47.1% of $\Delta E_{\text{crude steel}}$;
- $E_{\text{heat recovery}} = -29.3 \times 86.741 \div 1000 = -2.542\ \text{GJ/t}_{\text{crude steel}}$, accounting for -95.2% of $\Delta E_{\text{crude steel}}$.

2) the primary energy consumption per tonne of CO_2 reduced:

$$E_{CO_2} = \frac{\Delta E_{\text{crude steel}}}{(EOC)_{\text{reference}} - (EOC)_{\text{retrofit}}} = \frac{E_{CO_2\ \text{separation}} + E_{O_2\ \text{separation}} + E_{CO_2\ \text{compression}} - E_{\text{lime kiln}} - E_{\text{heat recovery}}}{M_{CO_2}^{\text{reference}} - (1-\eta_{\text{capt.}}) \cdot M_{CO_2}^{\text{retrofit}}}$$
$$= \frac{2.671}{1.800 - 1.492 \times \left(1 - \frac{0.362}{0.829}\right)} = 2.784\ \text{GJ/t}_{CO_2}$$

When we further break this energy consumption, E_{CO_2} , down,

- $$E'_{CO_2\ \text{separation}} = \frac{E_{CO_2\ \text{separation}}}{M_{CO_2}^{\text{reference}} - (1-\eta_{\text{capt.}}) \cdot M_{CO_2}^{\text{retrofit}}} = \frac{4.703}{1.800 - 1.492 \times \left(1 - \frac{0.362}{0.829}\right)} = 4.904\ \text{GJ/t}_{CO_2}$$,

  accounting for 176.1% of the total energy consumption, E_{CO_2};
- $$E'_{O_2\ \text{separation}} = \frac{E_{O_2\ \text{separation}}}{M_{CO_2}^{\text{reference}} - (1-\eta_{\text{capt.}}) \cdot M_{CO_2}^{\text{retrofit}}} = \frac{0.662}{1.800 - 1.492 \times \left(1 - \frac{0.362}{0.829}\right)} = 0.690\ \text{GJ/t}_{CO_2}$$,

  accounting for 24.8% of E_{CO_2};

- $E'_{CO_2\ compression}=\dfrac{E_{CO_2\ compression}}{M^{reference}_{CO_2}-\left(1-\eta_{capt.}\right)\cdot M^{retrofit}_{CO_2}}=\dfrac{1.106}{1.800-1.492\times\left(1-\dfrac{0.362}{0.829}\right)}=1.153\ \text{GJ/t}_{CO_2}$,

  accounting for 41.4% of E_{CO_2};

- $E'_{lime\ kiln}=\dfrac{E_{lime\ kiln}}{M^{reference}_{CO_2}-\left(1-\eta_{capt.}\right)\cdot M^{retrofit}_{CO_2}}=\dfrac{-1.258}{1.800-1.492\times\left(1-\dfrac{0.362}{0.829}\right)}=-1.312\ \text{GJ/t}_{CO_2}$,

  accounting for -47.1% of E_{CO_2};

- $E'_{heat\ recovery}=\dfrac{E_{heat\ recovery}}{M^{reference}_{CO_2}-\left(1-\eta_{capt.}\right)\cdot M^{retrofit}_{CO_2}}=\dfrac{-2.542}{1.800-1.492\times\left(1-\dfrac{0.362}{0.829}\right)}=-2.651\ \text{GJ/t}_{CO_2}$,

  accounting for -95.2% of E_{CO_2}.

<u>CO_2 reduction:</u>

<u>1) CO_2 capture efficiency in the add-on kiln of the CaL-LP scheme:</u>

$$\eta_{capt.}=\frac{0.362}{\beta}\times100\%=\frac{0.362}{0.829}\times100\%=43.667\%$$

<u>2) the total amount of CO_2 reduced per tonne of crude steel produced:</u>

$$\Delta M_{CO_2}=M^{reference}_{CO_2}-\left(1-\eta_{capt.}\right)\cdot M^{retrofit}_{CO_2}=1.800-1.492\times\left(1-\frac{0.362}{0.829}\right)=0.959\ \text{t}_{CO_2}/\text{t}_{crude\ steel}$$

When we further break this amount of CO_2 reduced, ΔM_{CO_2} , down,

- Amount of CO_2 reduced from the add-on kiln of the CaL-LP scheme:

  $\Delta M^{add\text{-}on\ kiln}_{CO_2}=\eta_{capt.}\cdot M^{retrofit}_{CO_2}=43.667\%\times1.492=0.651\ \text{t}_{CO_2}/\text{t}_{crude\ steel}$

- Amount of CO_2 reduced from the lime kiln of the CaL-LP scheme:

  $\Delta M^{lime\ kiln}_{CO_2}=\dfrac{MW_{CO_2}}{MW_{CaCO_3}}\cdot M_{limestone}+\dfrac{2.54\times E_{lime\ kiln}}{29.3}=\dfrac{44}{100}\times0.3+\dfrac{2.54\times1.258}{29.3}=0.241\ \text{t}_{CO_2}/\text{t}_{crude\ steel}$

- Amount of CO_2 reduced due to the net electricity recovered from the CaL-LP scheme:

  $\Delta M^{electricity}_{CO_2}=M^{heat\ recovery}_{CO_2}-M^{O_2\ separation}_{CO_2}-M^{CO_2\ compression}_{CO_2}$
  $=\left(220.322-57.406-95.842\right)\div1000=0.067\ \text{t}_{CO_2}/\text{t}_{crude\ steel}$

<u>3) overall CO_2 reduction efficiency:</u>

$$\varphi_{CO_2} = \frac{M_{CO_2}^{\text{reference}} - (1-\eta_{\text{capt.}}) \cdot M_{CO_2}^{\text{retrofit}}}{M_{CO_2}^{\text{reference}}} \times 100\% = \frac{1.800 - 1.492 \times \left(1 - \frac{0.362}{0.829}\right)}{1.800} \times 100\% = 53.306\%$$

When we further break the overall CO_2 reduction efficiency, φ_{CO_2} , down,

- CO_2 reduction efficiency from the add-on kiln of the CaL-LP scheme:

$$\varphi_{CO_2}^{\text{add-on kiln}} = \frac{\Delta M_{CO_2}^{\text{add-on kiln}}}{\Delta M_{CO_2}} \times 100\% = \frac{0.651}{0.959} \times 100\% = 67.9\%$$

- CO_2 reduction efficiency from the lime kiln of the CaL-LP scheme:

$$\varphi_{CO_2}^{\text{lime kiln}} = \frac{\Delta M_{CO_2}^{\text{lime kiln}}}{\Delta M_{CO_2}} \times 100\% = \frac{0.241}{0.959} \times 100\% = 25.1\%$$

- CO_2 reduction efficiency due to the net electricity recovered from the CaL-LP scheme: $\varphi_{CO_2}^{\text{electricity}} = \frac{\Delta M_{CO_2}^{\text{electricity}}}{\Delta M_{CO_2}} \times 100\% = \frac{0.067}{0.959} \times 100\% = 7.0\%$

<u>Cost of steel production and CO_2 reduction:</u>

<u>1) the incremental cost of crude steel:</u>

$$\Delta C_{\text{crude steel}} = (COS)_{\text{retrofit}} - (COS)_{\text{reference}} = \alpha \cdot \Delta I' + \Delta C'_{\text{Energy}} + \Delta C'_{\text{O\&M}} + \Delta C'_{\text{Material}}$$
$$= 0.11 \times (45+25) + 1.5 \times 2.671 + 4\% \times (45+25) + 0 = 14.51\ €_{2010}/\text{t}_{\text{crude steel}}$$

When we further break the incremental cost of crude steel, $\Delta C_{\text{crude steel}}$, down,

- Cost contribution from the incremental capital requirement due to implementation of the CaL-LP scheme:

$$\Delta C_{\text{crude steel}}^{\text{capital}} = \alpha \cdot \Delta I' = 0.11 \times (45+25) = 7.70\ €_{2010}/\text{t}_{\text{crude steel}}$$
$$\omega_{\text{capital}} = \frac{\Delta C_{\text{crude steel}}^{\text{capital}}}{\Delta C_{\text{crude steel}}} \times 100\% = \frac{7.70}{14.51} \times 100\% = 53.1\%$$

- Cost contribution from the incremental cost of energy due to implementation of the CaL-LP scheme:

$$\Delta C_{\text{crude steel}}^{\text{energy}} = \Delta C'_{\text{Energy}} = EP \cdot \Delta E_{\text{crude steel}} = 1.5 \times 2.671 = 4.01\ €_{2010}/\text{t}_{\text{crude steel}}$$
$$\omega_{\text{energy}} = \frac{\Delta C_{\text{crude steel}}^{\text{energy}}}{\Delta C_{\text{crude steel}}} \times 100\% = \frac{4.01}{14.51} \times 100\% = 27.6\%$$

- Cost contribution from the incremental operation and maintenance (O&M) costs due to implementation of the CaL-LP scheme:

$$\Delta C^{\text{O\&M}}_{\text{crude steel}} = \Delta C^{'}_{\text{O\&M}} = \mu \cdot \Delta I^{'} = 4\% \times (45 + 25) = 2.80\ €_{2010}/\text{t}_{\text{crude steel}}$$

$$\omega_{\text{O\&M}} = \frac{\Delta C^{\text{O\&M}}_{\text{crude steel}}}{\Delta C_{\text{crude steel}}} \times 100\% = \frac{2.80}{14.51} \times 100\% = 19.3\%$$

- Cost contribution from the incremental cost of raw materials due to implementation of the CaL-LP scheme is almost zero, since no additional material consumption is required.

2) CO_2 avoidance cost:

$$AC = \frac{\alpha \cdot \Delta I^{'} + \Delta C^{'}_{\text{Energy}} + \Delta C^{'}_{\text{O\&M}} + \Delta C^{'}_{\text{Material}}}{(EOC)_{\text{reference}} - (EOC)_{\text{retrofit}}} = \frac{\Delta C_{\text{crude steel}}}{\Delta M_{\text{CO}_2}} = \frac{14.51\ €_{2010}/\text{t}_{\text{crude steel}}}{0.959\ \text{t}_{\text{CO}_2}/\text{t}_{\text{crude steel}}} = 15.13\ €_{2010}/\text{t}_{\text{CO}_2}$$

When we further break the CO_2 avoidance cost, *AC*, down,

- Cost contribution from the incremental capital requirement due to implementation of the CaL-LP scheme:

$$AC_{\text{capital}} = \frac{\alpha \cdot \Delta I^{'}}{\Delta M_{\text{CO}_2}} = \frac{0.11 \times (45 + 25)}{0.959} = 8.03\ €_{2010}/\text{t}_{\text{CO}_2}$$

$$\varpi_{\text{capital}} = \frac{AC_{\text{capital}}}{AC} \times 100\% = \frac{8.03}{15.13} \times 100\% = 53.1\%$$

- Cost contribution from the incremental cost of energy due to implementation of the CaL-LP scheme:

$$AC_{\text{energy}} = \frac{EP \cdot \Delta E_{\text{crude steel}}}{\Delta M_{\text{CO}_2}} = \frac{1.5 \times 2.671}{0.959} = 4.18\ €_{2010}/\text{t}_{\text{CO}_2}$$

$$\varpi_{\text{energy}} = \frac{AC_{\text{energy}}}{AC} \times 100\% = \frac{4.18}{15.13} \times 100\% = 27.6\%$$

- Cost contribution from the incremental operation and maintenance (O&M) costs due to implementation of the CaL-LP scheme:

$$AC_{\text{O\&M}} = \frac{\mu \cdot \Delta I^{'}}{\Delta M_{\text{CO}_2}} = \frac{4\% \times (45 + 25)}{0.959} = 2.92\ €_{2010}/\text{t}_{\text{CO}_2}$$

$$\omega_{\text{O\&M}} = \frac{AC_{\text{O\&M}}}{AC} \times 100\% = \frac{2.92}{15.13} \times 100\% = 19.3\%$$

- Cost contribution from the incremental cost of raw materials due to implementation of the CaL-LP scheme is almost zero, since no additional material consumption is required.

Supplementary References

58. Grasa, G. S. & Abanades, J. C. CO_2 capture capacity of CaO in long series of carbonation–calcination cycles. *Ind. Eng. Chem. Res.* **45,** 8846–8851 (2006).

59. Li, Z., Cai, N. & Croiset, E. Process analysis of CO_2 capture from flue gas using carbonation–calcination cycles. *AICHE J.* **54,** 1912–1925 (2008).

60. Abanades, J. C. & Alvarez, D. Conversion limits in the reaction of CO_2 with lime. *Energy Fuels* **17,** 308–315 (2003).

61. Wang, J. & Anthony, E. J. On the decay behavior of the CO_2 absorption capacity of CaO-based sorbents. *Ind. Eng. Chem. Res.* **44,** 627–629 (2005).

62. Tu, H. & Liu, C. Calculation of CO_2 emission of standard coal. *Coal Qual. Technol.* **2,** 57–60 (2014).

63. Zhao, M., Minett, A. I. & Harris, A. T. A review of techno-economic models for the retrofitting of conventional pulverised-coal power plants for post-combustion capture (PCC) of CO_2. *Energy Environ. Sci.* **6,** 25–40 (2013).

64. Abanades, J. C. *et al.* Cost structure of a postcombustion CO_2 capture system using CaO. *Environ. Sci. Technol.* **41,** 5523−5527 (2007).

65. Dean, C. C., Blamey, J., Florin, N. H., Al-Jeboori, M. J. & Fennell, P. S. The calcium looping cycle for CO_2 capture from power generation, cement manufacture and hydrogen production. *Chem. Eng. Res. Des.* **89,** 836–855 (2011).
